# Automatic detection of mesiodens on panoramic radiographs using artificial intelligence

**DOI:** 10.1038/s41598-021-02571-x

**Published:** 2021-11-29

**Authors:** Eun-Gyu Ha, Kug Jin Jeon, Young Hyun Kim, Jae-Young Kim, Sang-Sun Han

**Affiliations:** 1 Department of Oral and Maxillofacial Radiology, Yonsei University College of Dentistry, 50-1 Yonsei-ro Seodaemun-gu, Seoul, 03722 South Korea; 2 Department of Oral and Maxillofacial Surgery, Gangnam Severance Hospital, Yonsei University College of Dentistry, Seoul, South Korea

**Keywords:** Dental diseases, Dentistry, Panoramic radiography, Mathematics and computing

## Abstract

This study aimed to develop an artificial intelligence model that can detect mesiodens on panoramic radiographs of various dentition groups. Panoramic radiographs of 612 patients were used for training. A convolutional neural network (CNN) model based on YOLOv3 for detecting mesiodens was developed. The model performance according to three dentition groups (primary, mixed, and permanent dentition) was evaluated, both internally (130 images) and externally (118 images), using a multi-center dataset. To investigate the effect of image preprocessing, contrast-limited histogram equalization (CLAHE) was applied to the original images. The accuracy of the internal test dataset was 96.2% and that of the external test dataset was 89.8% in the original images. For the primary, mixed, and permanent dentition, the accuracy of the internal test dataset was 96.7%, 97.5%, and 93.3%, respectively, and the accuracy of the external test dataset was 86.7%, 95.3%, and 86.7%, respectively. The CLAHE images yielded less accurate results than the original images in both test datasets. The proposed model showed good performance in the internal and external test datasets and had the potential for clinical use to detect mesiodens on panoramic radiographs of all dentition types. The CLAHE preprocessing had a negligible effect on model performance.

## Introduction

Mesiodens refers to a supernumerary tooth located in the anterior maxilla, and it is the most common type of supernumerary tooth^1^. Impacted mesiodens has various effects on the succeeding teeth or adjacent permanent teeth and it causes delayed eruption, rotation, displacement, crowding, diastema, and root resorption^2^. Odontogenic cysts involving mesiodens may also occur, interfering with implant placement and orthodontic tooth movement^3^.

Panoramic radiography is a widely used imaging modality for diagnosis and treatment planning in dentistry^4–6^. However, the geometry of panoramic radiography results in disadvantages such as image blurring, distortion, low resolution, and superposition of additional structures, and these factors make it difficult to accurately diagnose lesions^7–10^. The anterior regions of the maxilla and mandible are especially difficult to diagnose due to the thin image layer of the device, overlapping of the vertebrae^11^, and the air space between the tongue and the palate^12^. The degree of overlapping and blurring depends on the device and the patient’s position. Thus, for a clinician with little experience or when many images need to be read in a short time, the detection of mesiodens on panoramic radiographs is often missed.

Because mesiodens may cause a variety of complications, its diagnosis is important. An accurate diagnosis would allow timely removal of mesiodens, reducing the risk of complications, the risk of which is especially high in mixed and primary dentition due to overlap between the developing succeeding tooth germ and mesiodens. Thus, it is helpful for dental clinicians to develop an automatic diagnostic artificial intelligence (AI) model using panoramic radiography. However, only one study has focused on the automated detection of mesiodens, and it was limited to permanent dentition^13^. Image preprocessing has been widely applied in medical image-based deep learning studies^14–16^. Contrast-limited histogram equalization (CLAHE) has often been used in studies using panoramic radiography^17,18^, but no previous studies have focused on comparing the model performance between original images and CLAHE images.

This study aimed to develop an AI model to automatically detect mesiodens on panoramic radiography for primary, mixed, and permanent dentition groups. The performance of the model was validated internally and externally with multi-center test data, and the effect of preprocessing was investigated.

## Materials and methods

### Subjects

This study was approved by the Institutional Review Board of Yonsei University Dental Hospital (No. 2-2021-0043) and was conducted in accordance with ethical regulations and guidelines. The requirement for informed consent was waived since this was a retrospective study and all data in this study were used after anonymization.

The training and validation of the AI model used the panoramic radiographs of 612 patients with an impacted mesiodens in the anterior maxillary region who visited Yonsei University Dental Hospital from July 2017 to January 2021. The panoramic images were acquired from two types of equipment: RAYSCAN Alpha (Ray Co., Ltd., Hwaseong-si, Korea) and PaX-i3D Green (Vatech Co., Ltd., Hwaseong-si, Korea). The panoramic images were divided into three groups according to the stage of tooth development, as follows: primary dentition (3–6 years), mixed dentition (7–13 years), and permanent dentition (over 14 years old). Table 1 shows the detailed distribution of the training and validation dataset. Since mesiodens is more difficult to diagnose in primary and mixed dentition than in permanent dentition, panoramic radiographs were collected mainly with primary and mixed dentition.

**Table 1:** Number of panoramic radiographs with mesiodens in the training and validation datasets.

Group	Training	Validation	Total
Primary dentition	267	29	296
Mixed dentition	186	21	207
Permanent dentition	98	11	109
Total	551	61	612

The test dataset consisted of internal data from Yonsei University Dental Hospital and external data obtained using ProMax (Planmeca Inc., Helsinki, Finland) from Gangnam Severance Hospital. The internal test dataset consisted of 65 images with mesiodens and 65 images without mesiodens, and the external test dataset consisted of 58 images with mesiodens and 60 images without mesiodens. Table 2 shows the test dataset. All panoramic images were selected from patients who had cone-beam computed tomography scans, which were used to confirm the presence of mesiodens.

**Table 2:** Number of panoramic radiographs in the internal and external test datasets.

Group	Internal test		External test	
	With mesiodens	Without mesiodens	With mesiodens	Without mesiodens
Primary dentition	30	30	10	20
Mixed dentition	20	20	23	20
Permanent dentition	15	15	25	20
Total	65	65	58	60

### Image preparation and preprocessing

Panoramic radiographs were downloaded in the bitmap format with a matrix size of approximately 2996 (width) × 1502 (height) pixels. As preprocessing, the CLAHE method was applied to all the original images. The CLAHE method involves applying equalization based on dividing the image into several regions of almost equal sizes^19^. Applying the CLAHE method has been found to improve the image quality compared to other histogram equalization methods, and it has been widely used in deep learning model studies based on medical images^20–23^. The experiments using the original images and CLAHE images were implemented in Windows 10 with the TensorFlow 1.16.0 framework on an NVIDIA GPU (TITAN RTX).

### Development and evaluation of the model

**Figure 1:**
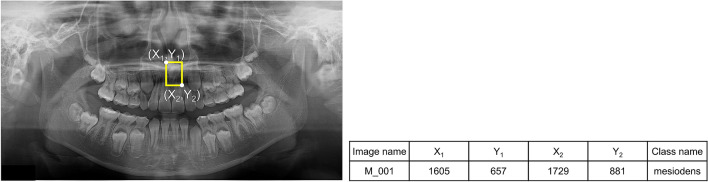
The oral radiologist annotated the mesiodens with a yellow rectangular box. The coordinate information of the upper left (X_1_, Y_1_) and the lower right (X_2_, Y_2_) was determined, and the class name was extracted as “mesiodens.”

We developed a model based on YOLOv3 for detecting mesiodens. YOLO is a representative deep learning (DL) detection algorithm that has shown much better performance than other detection algorithms^24^. This model required information on the training dataset (i.e., the location and class name of ground truth) for model training. An oral radiologist with over 20 years of experience performed annotation using a rectangular region of interest (ROI) including just the mesiodens as a gold standard using the graphical image annotation tool LabelImg (version 1.8.4, available at https://github.com/tzutalin/labelImg). From the annotations, the coordinates of the upper left (X_1_, Y_1_) and lower right (X_2_, Y_2_) corners of the ROI were determined and its class name was extracted as “mesiodens” (Fig. 1). The information extracted from the input images was used in the model training process.

**Figure 2:**
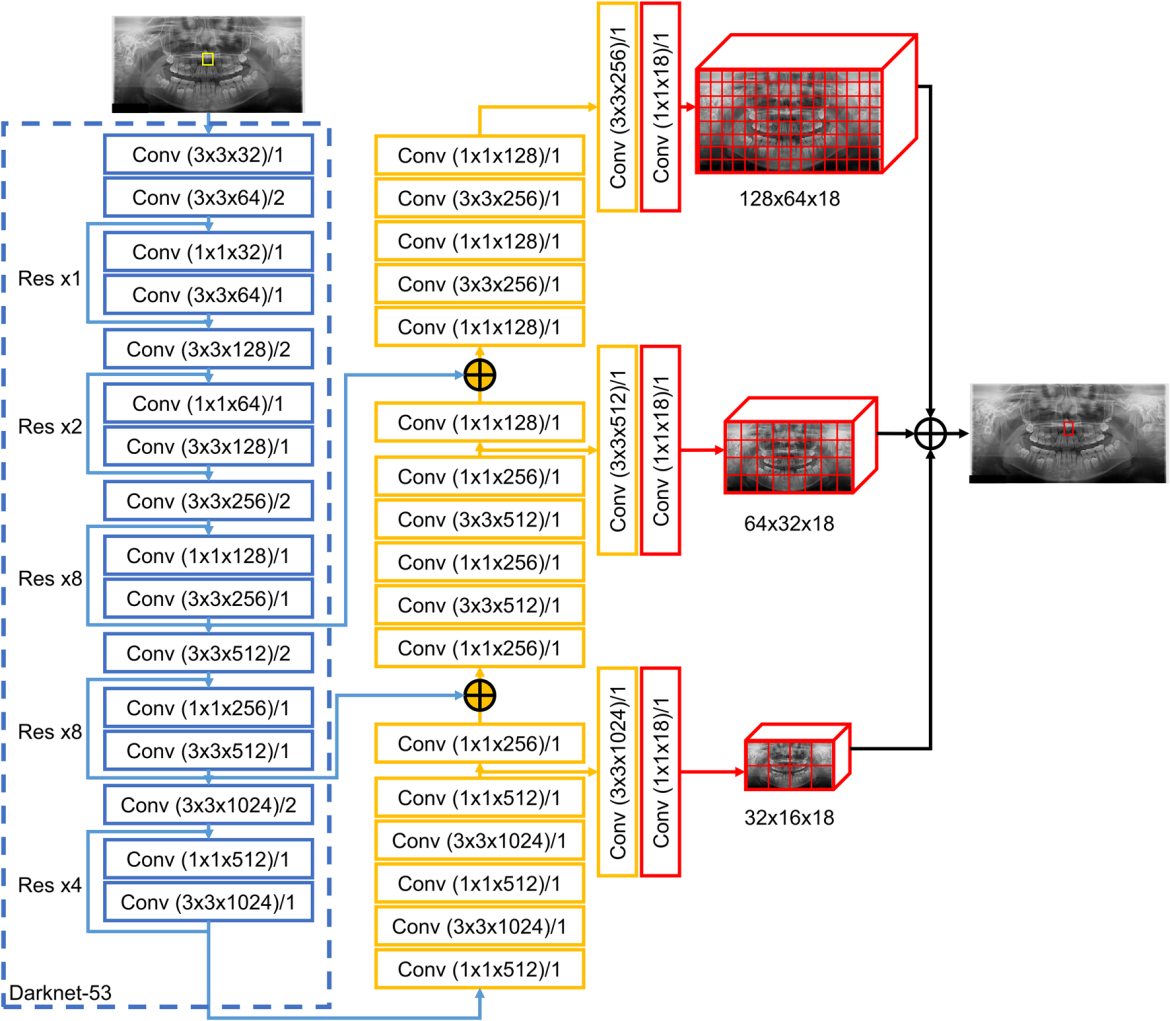
Overall architecture of the proposed model from YOLOv3. Res, residual network; Conv, convolutional network.

The panoramic images were resized to 512 (width) × 256 (height) pixels and input to the backbone (darknet-53 architecture), which consisted of 53 convolutional layers, with batch normalization added to all convolutional layers. The leak rectified linear unit (ReLU) was used as the activation function. The output comprised three feature maps that passed through the convolutional layers, and mesiodens was automatically detected at different resolutions through these three feature maps. When the model detected mesiodens, it provided an image marked with a red box in the detected area, and if there was no detected mesiodens, it provided the input panoramic image without a box (Fig. 2). It was judged that the model correctly predicted mesiodens when the intersection over union (IoU) value of the detected mesiodens area was 0.5 or higher^25^. The first step of model training used the weights of the YOLO model pre-trained using the COCO dataset^24^ and the model was trained for 300 epochs with our dataset. In the initial 150 epochs, only the weights of the last 3 layers were trained with our dataset, and in the next 150 epochs, the weights of the entire network were trained on our dataset.

In the original images, the detection performance of the proposed model was evaluated in terms of accuracy, sensitivity, and specificity using internal and external test datasets. The results with and without the CLAHE method were compared for the primary, mixed, and permanent groups.

## Results

Table 3 shows the accuracy, sensitivity and specificity of the model using internal and external test datasets for the original images. The accuracy of the internal test dataset was 96.2% and that of the external test dataset was 89.8%. For the primary, mixed, and permanent dentition, the accuracy of the internal test dataset was 96.7%, 97.5%, and 93.3%, respectively, and the accuracy of the external test dataset was 86.7%, 95.3%, and 86.7%, respectively.

**Table 3:** Accuracy, sensitivity, and specificity of the model (%).

	Internal test			External test		
	Accuracy	Sensitivity	Specificity	Accuracy	Sensitivity	Specificity
Primary	96.7	96.7	96.7	86.7	90.0	85.0
Mixed	97.5	95.0	100.0	95.3	95.7	95.0
Permanent	93.3	93.3	93.3	86.7	80.0	95.0
Total	96.2	95.4	96.9	89.8	87.9	91.7

**Figure 3:**
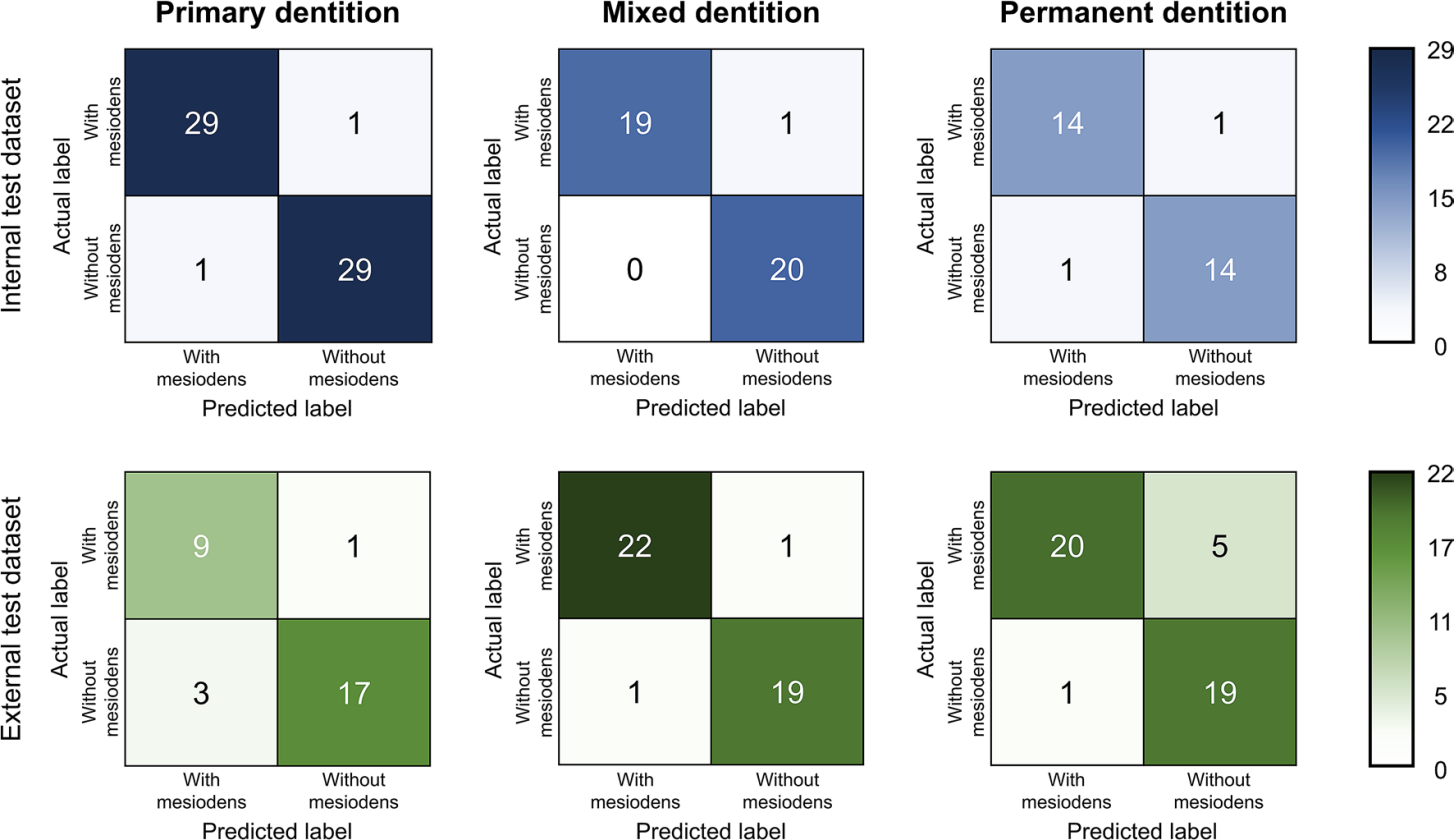
Confusion matrices of the internal and external test dataset for the primary, mixed, and permanent dentition groups.

Confusion matrices of the model using the internal and external test datasets according to the dentition group are shown in Fig. 3.

Table 4 shows the accuracy, sensitivity, and specificity of the model using internal and external test datasets depending on whether the CLAHE preprocessing method was applied. The accuracy and specificity of the original images was higher than that of the CLAHE images for both the internal and external test datasets.

**Table 4:** Accuracy, sensitivity, and specificity of the model according to CLAHE preprocessing (%).

	Internal test		External test	
	Original image	CLAHE image	Original image	CLAHE image
Accuracy	96.2	93.1	89.8	88.1
Sensitivity	95.4	95.4	87.9	86.2
Specificity	96.9	90.8	91.7	90.0

**Figure 4:**
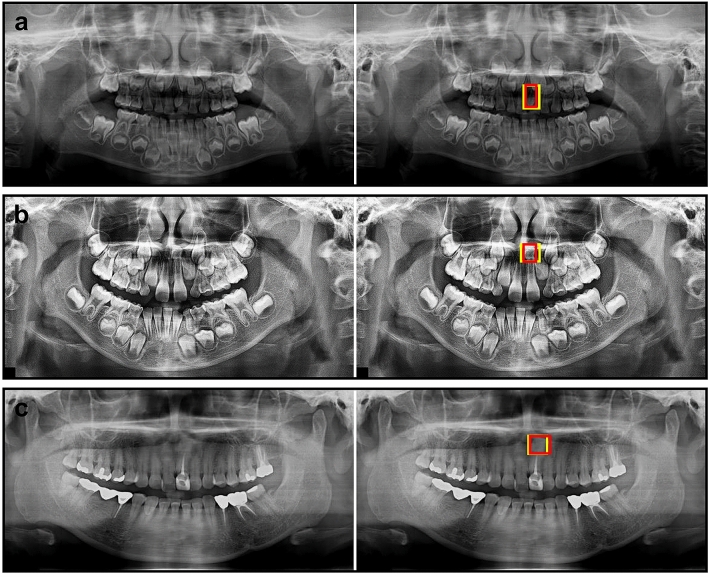
Examples of correctly detected mesiodens in primary dentition (**a**), mixed dentition (**b**), and permanent dentition (**c**). The left side was the input image and the right side was the output image. The mesiodens annotated by the radiologist is shown as the yellow box and the automatically detected mesiodens is shown as the red box.

**Figure 5:**
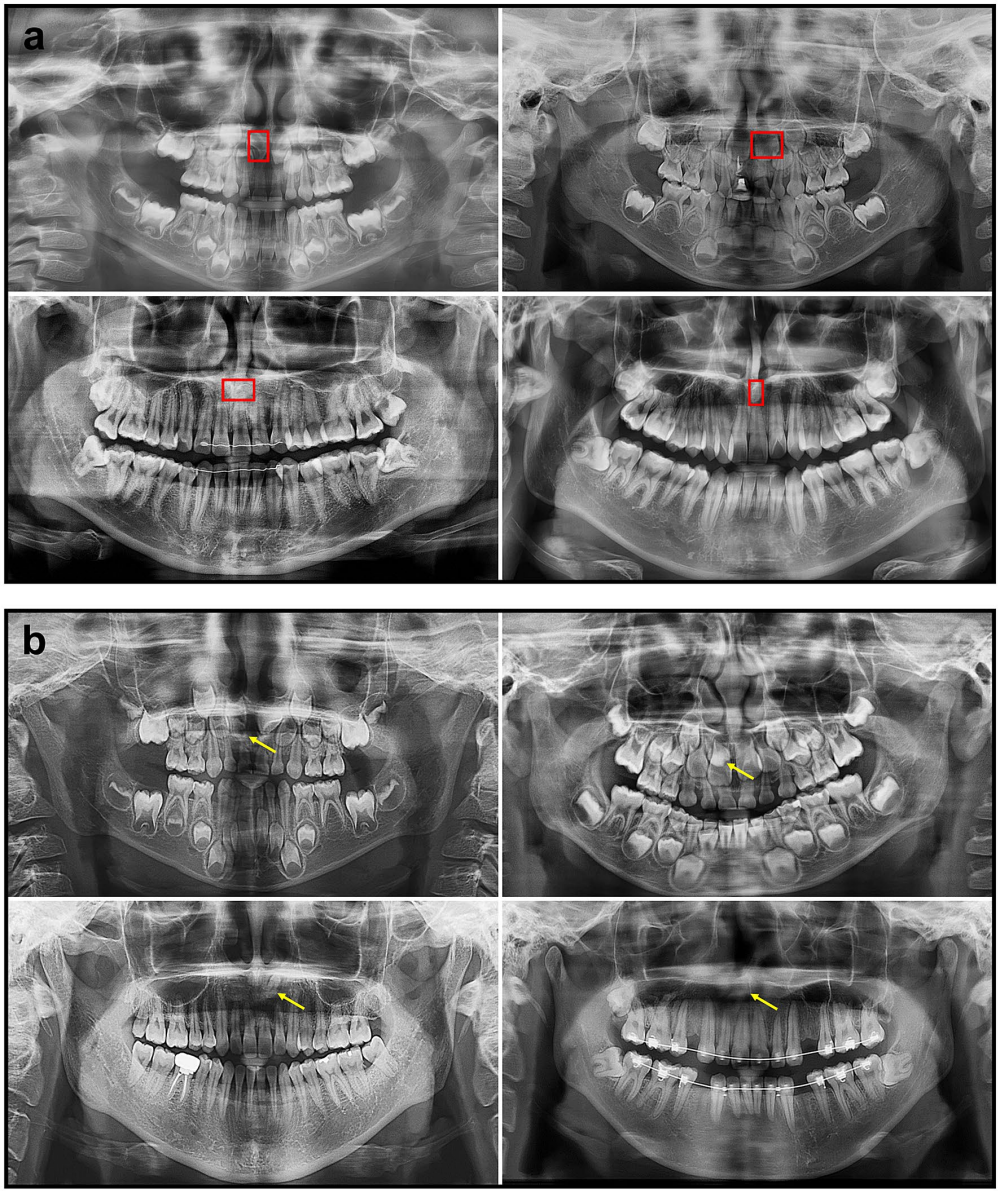
Examples of false-positive cases (**a**) and false-negative cases (**b**). Red boxes denote the incorrectly detected regions, while yellow arrows show undetected mesiodens.

Figure 4 shows examples of mesiodens correctly detected by the developed model. False-positive and false-negative cases are presented in Fig. 5. The incorrect detection cases were confused with the succeeding tooth germ, anterior nasal spine, and ala of the nose.

## Discussion

AI research has been conducted in a diverse range of fields, and numerous studies have also been conducted in the dental field. DL algorithms with convolutional neural networks (CNNs) have received considerable attention and have been used for segmentation, classification, and detection with panoramic radiographs^17,21,26^. Many studies have applied these techniques to orthodontic diagnoses, root canal treatment, tooth extraction, and the diagnosis of lesions^27,28^. The most common application has been for classifying cysts and tumors of the jaw^17,29,30^.

Recently, various algorithms such as the YOLO algorithm, deformable parts model, and R-CNN algorithm have been introduced and found to effectively detect cancer, nodules, fractures, or other lesions on medical images^31–34^. The YOLO algorithm was first proposed by Redmon et al.^24^, and it has been applied in the dental field to detect various diseases on panoramic radiographs^17,29,35,36^. Yang et al.^35^ detected cysts and tumors of the jaw using YOLOv2 and obtained a precision of 0.707 and recall of 0.680. Kwon et al.^17^ developed a YOLOv3 model that showed high performance, with sensitivity values of 91.4%, 82.8%, 98.4%, and 71.7% for dentigerous cysts, periapical cysts, odontogenic keratocysts, and ameloblastomas, respectively. Son et al.^36^ developed a model to detect mandibular fractures using YOLOv4.

Early AI research tended to use images obtained from a single device at one institution^37–39^, making the resulting models difficult to generalize, and the problem arose that performance deteriorated when AI models were applied in actual clinical practice. The assessment of the real-world clinical performance of a model-based DL algorithm requires external validation using data collected at institutions other than the institution that provided the training data^40^. In particular, multi-center research is especially important for panoramic radiography because the thickness of the image layer is different for each type of equipment, and blurring and overlapping can vary depending on the equipment and patient position. We conducted training and validation using images obtained with two types of equipment, and tested the model with images obtained from different devices at internal and external institutions to confirm generalizability. The accuracy, sensitivity, and specificity of the developed model using the internal test dataset were all more than 95%, and the corresponding performance metrics with the external test dataset showed only slightly poorer performance compared with the internal test results, with values of 89.8%, 87.9%, and 91.7%, respectively. Previous studies that performed external testing generally showed similar trends. In a DL study for the diagnosis of mandibular condylar fractures using panoramic radiographs from two hospitals, when images from the same hospital were used as the training and test data sets, the accuracy was 80.4% and 81%, respectively. In contrast, when images from different hospitals were used, the accuracy decreased to 59.0% and 60%^21^. This drop-off in performance is thought to be due to variation between the training panoramic radiographs and the external panoramic radiographs (e.g., differences in image noise and brightness), as mentioned in other research^41^.

Due to the geometric configuration of panoramic radiographs, the maxillary anterior region is the most blurred, and diseases or mesiodens in this area is often missed. Kuwada et al.^13^ developed DL models (AlexNet, VGG-16, and DetectNet) and compared their performance. A limitation of that previous study is that it was performed on permanent dentition only, with panoramic radiographs from one institution, and without external testing. In contrast, our study developed a model using the YOLOv3 algorithm and compared its performance according to preprocessing. Furthermore, our study was conducted with all dentition groups (primary, mixed, and permanent dentition) on panoramic radiographs and was tested internally and externally using multi-center data. Our proposed model showed high accuracy (more than 93%) in primary and mixed dentition, as well as permanent dentition, and the accuracy, sensitivity, and specificity showed good performance (over 87%) for the external test dataset. The CLAHE preprocessing method was also applied to investigate whether preprocessing improved model performance. Rahman et al.^42^ studied five image enhancement techniques, including CLAHE, to detect COVID-19 on chest X-rays, all of which showed very reliable performance. Some studies have applied CLAHE to panoramic radiography^17,18^. In the present study, the original images without CLAHE had higher accuracy, sensitivity, and specificity, both internally and externally, except for sensitivity in internal testing. Therefore, the CLAHE preprocessing method had a negligible effect on the performance of the model, and preprocessing should be carefully considered during model development.

Our study has limitations in that the number of samples used was small and that multiple mesiodens were not included. Although it is currently very challenging to collect external data related to this topic, further research using imaging data from more centers and devices will improve the performance of the model.

## Conclusion

The developed CNN model for fully automatic detection of mesiodens showed high performance in multi-center tests for all types of dentition, including primary, mixed, and permanent dentition. The developed model has the potential to help dental clinicians diagnose mesiodens on panoramic radiographs.

## Data Availability

The data generated and analyzed during the current study are not publicly available due to privacy laws and policies in Korea, but are available from the corresponding author on reasonable request.
